# Artificial Intelligence in Post-Liver Transplantation: A Scoping Review of Comparative Model Performance

**DOI:** 10.3390/jcm15041491

**Published:** 2026-02-13

**Authors:** Ileana Lulic, Ivan Gornik, Jadranka Pavicic Saric, Dunja Rogic, Alberto Gallego, Laura Karla Bozic, Nikola Prpic, Iva Bacak Kocman, Gorjana Erceg, Jelena Pegan, Iva Majurec, Damira Vukicevic Stironja, Lucija Ermacora, Lorka Tarnovski, Stipislav Jadrijevic, Danko Mikulic, Filip Jadrijevic, Lana Mihanovic, Dinka Lulic

**Affiliations:** 1 Solid Organ Transplant Unit, Department of Anesthesiology, Intensive Care and Pain Medicine, Clinical Hospital Merkur, Zajceva 19, 10 000 Zagreb, Croatia; jadranka.pavicic.saric@kb-merkur.hr (J.P.S.); blaura210@gmail.com (L.K.B.); nikolaprpic2@gmail.com (N.P.); bacakkocmaniva@gmail.com (I.B.K.); gorjanaerceg@gmail.com (G.E.); jelenazenko@gmail.com (J.P.); ivamajurec@gmail.com (I.M.); vdamira@yahoo.com (D.V.S.); 2 Department of Medical Biochemistry and Hematology, Faculty of Pharmacy and Biochemistry, University of Zagreb, A. Kovacica 1, 10 000 Zagreb, Croatia; predstojnik.lab@kbc-zagreb.hr (D.R.); dinka.lulic@gmail.com (D.L.); 3 Department of Emergency Medicine, University Hospital Centre Zagreb, Kispaticeva 12, 10 000 Zagreb, Croatia; ivan.gornik@gmail.com; 4 Department of Laboratory Diagnostics, University Hospital Centre Zagreb, Kispaticeva 12, 10 000 Zagreb, Croatia; 5 Immediate Medical Care Unit, Saint James Hospital, SLM-1030 Sliema, Malta; albertongallego@gmail.com; 6 Solid Organ Transplant Unit, Department of Surgery, Clinical Hospital Merkur, Zajceva 19, 10 000 Zagreb, Croatia; ermacora00@gmail.com (L.E.); lorka.tarnovski@gmail.com (L.T.); stipislavjadrijevic@gmail.com (S.J.); mikulicdanko@gmail.com (D.M.); 7 School of Medicine, University of Mostar, Zrinskog Frankopana 34, 88000 Mostar, Bosnia and Herzegovina; fjadrijevic1@gmail.com; 8 School of Medicine, University of Zagreb, Salata 3, 10 000 Zagreb, Croatia; lana.mihanovic123@gmail.com

**Keywords:** artificial intelligence, liver transplantation, post-transplant care, clinical decision support, risk prediction

## Abstract

**Objective:** To map and characterize artificial intelligence (AI) applications in post-liver transplantation (LT) care, summarize comparative performance where available, and identify methodological and translational gaps. **Methods:** We conducted a scoping review in accordance with PRISMA-ScR. A comprehensive search of electronic databases was performed from inception through 1 April 2025. We included primary studies evaluating AI applications in the post-LT period (model development, validation, or implementation). Comparative studies were defined as those reporting head-to-head evaluation of at least two algorithmic models for the same task with quantitative performance metrics. Single-model studies were retained for evidence mapping but analyzed separately. Reviews and the other non-primary literature were included for contextual mapping. **Results:** The search yielded 3088 records. After deduplication, 2408 were screened, 191 full texts were assessed, and 65 studies were included. Of these, 52 reported primary outcome data. Clinical prediction studies (*n* = 43) focused on graft survival, rejection, fibrosis, oncologic recurrence, mortality, and composite outcomes. Operational studies (*n* = 3) evaluated early warning or bedside decision-support systems, and system-level studies (*n* = 6) examined benchmarking, donor–recipient matching, explainability, fairness, and cross-domain modeling. Most studies were retrospective and single-center, with internal validation commonly reported and external validation uncommon. **Conclusions:** AI research in post-LT care is expanding, with a predominant focus on clinical prediction. However, limited external validation, heterogeneous methods, and scarce real-world implementation constrain clinical readiness. Standardized evaluation and prospective integration are needed to determine whether AI tools can support decision-making and improve post-transplant outcomes.

## 1. Introduction

End-stage liver disease (ESLD) causes approximately two million deaths globally each year and remains a major contributor to premature mortality from cirrhosis and liver cancer [1]. Liver transplantation (LT) is the definitive treatment for ESLD and acute liver failure [2]. Advances in perioperative management, surgical techniques, immunosuppressive therapy, and antiviral treatment have improved early post-transplant outcomes, with one-year and five-year survival rates now exceeding 90% and 75%, respectively [3]. Despite these gains, LT is still associated with substantial morbidity and mortality. Post-transplant complications, particularly biliary and vascular events, remain common and contribute to graft dysfunction, reduced survival, and increased healthcare utilization [4]. Accurate risk stratification and early detection are therefore essential to preserve graft function and optimize long-term outcomes [5].

Post-transplant care is inherently data-intensive and requires continuous longitudinal monitoring of graft function, immunosuppressive exposure, laboratory trends, imaging findings, and evolving complications [6]. Although electronic health records and transplant registries provide structured documentation and enable benchmarking, post-LT data are often heterogeneous, incomplete, and inconsistently defined across centers. In addition, conventional statistical models and established prognostic scores may fail to capture complex non-linear interactions and time-dependent patterns that shape post-transplant trajectories. These limitations contribute to persistent gaps in individualized risk stratification and highlight the need for analytical approaches capable of integrating multimodal clinical data into clinically actionable predictions.

Artificial intelligence (AI) applications are increasingly explored across clinical medicine, including solid organ transplantation [7]. In LT recipients, AI has been evaluated to support risk prediction, complication detection, and outcome forecasting [8,9]. However, the evidence landscape is fragmented, with few studies providing head-to-head comparisons of AI model performance, optimization strategies, or clinical utility [10,11,12]. Moreover, many models lack external validation and are developed under heterogeneous methodological conditions, limiting their interpretability and clinical readiness. The expanding availability of digital health data highlights the need to map existing AI applications and clarify their methodological and translational maturity.

To date, no synthesis has specifically examined the comparative performance of AI applications in the post-LT setting. This scoping review aims to identify and characterize studies that directly compare AI models in post-transplant care for LT recipients, with a focus on methodological rigor, reported performance metrics, and clinical applicability.

## 2. Methods

### 2.1. Eligibility Criteria

This scoping review followed the Preferred Reporting Items for Systematic Reviews and Meta-Analyses extension for Scoping Reviews (PRISMA-ScR) guidelines [13]. We included primary studies evaluating AI applications developed for use in the post-LT phase, including model development, validation, or implementation. Comparative studies were defined as those reporting head-to-head evaluation of at least two distinct algorithmic models for the same post-LT task using the same dataset and outcome definition, with quantitative performance metrics. Comparators could include other AI/machine learning (ML) models, conventional statistical approaches (e.g., logistic regression or Cox regression), and/or established clinical scoring systems. Single-model development studies without a formal comparator were retained for evidence mapping but were analyzed separately from the comparative performance synthesis. The target population comprised adult or pediatric LT recipients receiving post-transplant care supported by AI applications.

Outcomes of interest were grouped into three domains: (1) clinical outcomes (e.g., graft survival, rejection, infection, readmission, and mortality), (2) operational outcomes (e.g., accuracy or timeliness of decision support, improvements in triage, or risk stratification), and (3) system-level outcomes (e.g., usability, explainability, fairness, benchmarking, donor–recipient matching, and other model performance measures).

Studies were eligible from database inception to 1 April 2025 in any language with an English abstract. We included randomized and non-randomized studies, prospective and retrospective cohort studies, technical validation studies, systematic and narrative reviews, methodological papers, and editorials. We excluded conference abstracts, protocols, unpublished or non-peer-reviewed materials, studies focused exclusively on preoperative or intraoperative phases of LT, animal or non-human studies, and studies comparing AI solely to clinical judgment without comparison to another algorithmic model (AI/ML, conventional statistical regression, or an established clinical scoring system).

### 2.2. Data Sources and Literature Search Approach

A comprehensive literature search was conducted in PubMed, Web of Science, and the Cochrane Central Register of Controlled Trials, covering studies published from database inception through 1 April 2025. Grey literature was not searched. The reference lists of all the included studies were screened manually to identify additional relevant publications. The search strategy was developed to identify studies evaluating AI applications in the post-transplantation phase of LT. Medical Subject Headings (MeSH) and free-text terms related to AI, ML, deep learning (DL), natural language processing (NLP), and LT were combined using Boolean operators. The complete search syntax is provided in the Supplementary Materials (Data S1).

### 2.3. Study Selection and Characteristics

Titles and abstracts were screened independently by two reviewers (I.L. and D.L.) after duplicate removal, followed by full-text assessment of potentially relevant articles. Discrepancies were resolved through discussion, and a third reviewer (D.R.) was consulted when consensus could not be reached. Reference management was performed using EndNote X9.

### 2.4. Data Extraction and Synthesis Procedures

Following the final selection of eligible studies, one reviewer (I.L.) developed a structured extraction spreadsheet tailored for this scoping review. Data were independently extracted and subsequently verified by a second reviewer (D.L.) for accuracy. Findings were synthesized through iterative team discussions to consolidate key points and guide thematic analysis. Discrepancies in interpretation were resolved through consensus.

## 3. Results

### 3.1. Study Selection and Overview of Included Evidence

**Figure 1:**
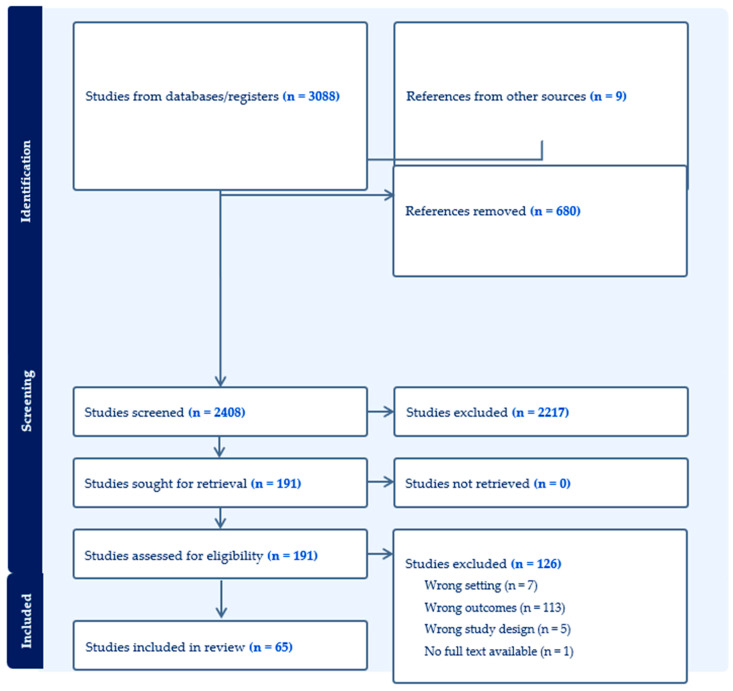
PRISMA flow diagram.

**Figure 2:**
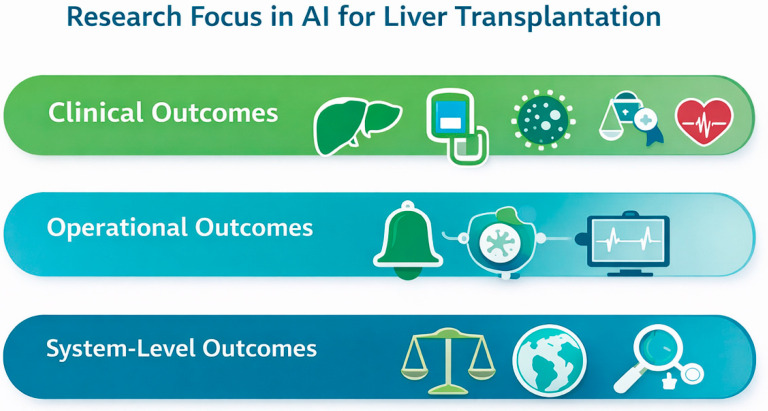
Conceptual domains of artificial intelligence applications in post-liver transplantation care. AI applications in post-liver transplantation care can be grouped into three domains: (1) clinical outcomes, representing models that predict graft or patient trajectories; (2) operational outcomes, referring to workflow-embedded tools such as early warning and decision-support systems; and (3) system-level outcomes, addressing population-level modeling, allocation, fairness, benchmarking, and deployment considerations.

The search identified 3088 records. After removing 680 duplicates, 2408 unique citations underwent title and abstract screening. Of these, 191 full-text articles were assessed for eligibility, and 65 studies were included in this scoping review [11,14,15,16,17,18,19,20,21,22,23,24,25,26,27,28,29,30,31,32,33,34,35,36,37,38,39,40,41,42,43,44,45,46,47,48,49,50,51,52,53,54,55,56,57,58,59,60,61,62,63,64,65,66,67,68,69,70,71,72,73,74,75,76,77]. The selection process is summarized in the PRISMA flow diagram (Figure 1). To contextualize the evidence landscape, AI applications identified in this scoping review were categorized into three conceptual domains based on their intended targets within post-transplant care: clinical outcomes, operational outcomes, and system-level outcomes (Figure 2). Of the 65 included studies, 52 reported primary outcome data and were included in thematic synthesis [14,15,16,17,18,19,20,21,22,23,24,25,26,27,28,29,30,31,32,33,34,35,36,37,38,39,40,41,42,43,44,45,46,47,48,49,50,51,52,53,54,55,56,57,58,59,60,61,62,63,64,65]. These comprised 43 studies categorized under Theme 1 (clinical outcomes) [14,15,16,17,18,19,20,21,22,23,24,25,26,27,28,29,30,31,32,33,34,35,36,37,38,39,40,41,42,43,44,45,46,47,48,49,50,51,52,53,54,55,56], consisting of one meta-analysis [14], 2 prospective studies [15,16], and 40 retrospective studies [17,18,19,20,21,22,23,24,25,26,27,28,29,30,31,32,33,34,35,36,37,38,39,40,41,42,43,44,45,46,47,48,49,50,51,52,53,54,55,56]; three retrospective studies addressing Theme 2 (operational outcomes) [57,58,59]; and six retrospective studies contributing to Theme 3 (system-level outcomes) [60,61,62,63,64,65]. The remaining 13 publications included four systematic reviews [66,67,68,69], seven narrative reviews [11,70,71,72,73,74,75], one methodological paper [76], and one commentary [77]. These were used to support contextual interpretation of methodological approaches and evidence gaps. Across the primary studies, internal validation approaches (e.g., split-sample testing or cross-validation) were common, whereas external validation using independent datasets was uncommon, and no studies reported prospective clinical validation or real-world implementation outcomes. Predicted endpoints varied in time horizon, spanning short-term (≤90 days), medium-term (3–12 months), and long-term (≥3 years) outcomes depending on the clinical target and available follow-up (Table 1, Table 2 and Table 3).

### 3.2. Theme 1: Clinical Outcomes

A summary of included studies evaluating AI-based prediction of clinical outcomes in post-LT care is provided in Table 1.

#### 3.2.1. Graft Survival and Rejection

Graft-related endpoints remain among the most clinically consequential challenges after LT. Accurately predicting graft survival, graft failure, rejection, fibrosis, and oncologic recurrence is essential for individualizing immunosuppression, allocating monitoring resources, and improving long-term outcomes. Of the 43 studies in Theme 1, fifteen specifically evaluated AI-driven models for graft-related endpoints, including graft survival, graft failure, rejection, fibrosis, or oncologic recurrence [14,18,20,23,24,27,28,33,35,43,44,46,48,50,55]. These investigations primarily applied ML approaches, including DL architectures, such as long short-term memory (LSTM) networks [18], multilayer perceptrons (MLPs) [46], and artificial neural networks (ANNs) [20,23,24,27,28,43,55]. Classical ML models were also widely utilized, including random survival forests (RSF) [33,44,50], gradient boosting techniques [44,48], and support vector machines (SVM) [35,44]. Several studies incorporated modern survival analysis frameworks, such as CoxNet and DeepSurv [33,50], while others leveraged transfer learning strategies [14] or integrated omics data [44] to enhance predictive performance. Most included studies directly compared multiple AI-driven models for post-LT outcome prediction, and many benchmarked them against traditional statistical methods and established clinical scoring systems, emphasizing their potential clinical utility [23,27,33,48,50]. Collectively, these studies address four major graft-related prediction tasks that are central to post-transplant care: (1) graft survival and failure prediction, (2) prediction of graft-compromising complications, (3) prediction of acute rejection, and (4) prediction of oncologic recurrence.

##### Graft Survival and Failure Prediction

Efforts to predict graft survival and failure after LT have progressed considerably over the past two decades. Early ANN-based models demonstrated limited discriminatory ability, achieving an area under the receiver operating characteristic curve (AUC-ROC) values of approximately 0.56 for short-term graft survival prediction [28]. Subsequent approaches incorporating perioperative and recipient-specific variables substantially improved performance. One such model reported an AUC-ROC of 0.8060, significantly outperforming established prognostic scores, including the model for end-stage liver disease (MELD), donor risk index (DRI), and balance of risk (BAR), which demonstrated respective AUC-ROC values of 0.50, 0.42, and 0.67 when compared against the ANN model (all *p* = 0.001) [23]. More recent investigations have leveraged DL architectures capable of modeling temporal patterns in clinical data. Weighted LSTM networks achieved an AUC-ROC of 0.798 (95%CI: 0.790–0.810), outperforming unweighted LSTM (AUC-ROC 0.761, 95%CI: 0.750–0.769, *p* = 0.031), recurrent neural network (RNN) (AUC-ROC 0.736, 95%CI: 0.721–0.744, *p* = 0.023), temporal convolutional network (TCN) (AUC-ROC 0.700, 95%CI: 0.662–0.747, *p* = 0.025), and random forest (RF) models (AUC-ROC 0.679, 95%CI: 0.652–0.707, *p* = 0.0081) [20]. Other ML-based approaches have targeted specific clinical contexts, including controlled donation after circulatory death using normothermic regional perfusion, where graft survival prediction at 3 and 12 months yielded AUC-ROC values of 0.82 and 0.83, respectively [24]. Models stratifying recipients by donor type (living versus deceased) also reported exceptional long-term graft survival prediction, with sensitivity, specificity, and accuracy exceeding 99% [48]. Finally, ANN models incorporating recipient comorbidities demonstrated improved discrimination of early graft loss, reporting a C-index of 0.745 (95%CI: 0.692–0.798, *p* < 0.001) [55].

##### Prediction of Graft-Compromising Complications

Multiple studies investigated AI-based strategies to anticipate post-transplant complications that jeopardize graft health, including fibrosis progression, biliary complications, and graft-versus-host disease (GVHD) [18,20,27,46]. In the context of fibrosis, ANN models trained on routine laboratory parameters achieved high accuracy, yielding an AUC-ROC of 0.93 (95%CI: 0.86–0.97), whereas logistic regression achieved an AUC-ROC of 0.84 (*p* = 0.045) [46]. DL methods that incorporated longitudinal clinical data further advanced this task. For example, LSTM networks attained an AUC-ROC of 0.798 (95%CI: 0.790–0.810) and surpassed both conventional ML algorithms and commonly used non-invasive fibrosis scores, such as the aspartate aminotransferase-to-platelet ratio index and the fibrosis-4 index (*p* < 0.05) [20]. Biliary injury was addressed by models integrating perioperative and recipient-specific variables, which demonstrated concordance index (C-index) values of 0.699 for biliary events and 0.784 for related mortality, indicating their capacity for risk-based stratification after LT [18]. GVHD, although uncommon, was similarly examined, with ML approaches achieving AUC-ROC values between 0.83 and 0.96, and thereby enabling earlier identification of this severe complication [27].

##### Prediction of Acute Rejection

Early neurocomputing models for predicting acute rejection following LT achieved moderate performance, with reported sensitivity between 65% and 75%, specificity ranging from 70% to 80%, and an overall accuracy of approximately 70–75%, consistently outperforming traditional linear models [43]. Subsequent efforts integrated immunogenetic markers with clinical variables, yielding substantial gains in predictive discrimination. In pediatric LT recipients, one such ML network achieved an AUC-ROC of 0.975 (95%CI: 0.96–0.99), with a sensitivity of 79.6%, specificity of 99.1%, and a correct classification rate of 97.1% [44]. More recently, a pan-organ ML framework incorporating transcriptomic data from liver, kidney, heart, and lung transplant cohorts demonstrated improved rejection prediction from peripheral blood samples. In LT recipients, this multi-organ model achieved an AUC-ROC of 0.71 compared to 0.55 for a liver-specific model, indicating enhanced generalizability when leveraging cross-organ molecular signatures [14].

##### Prediction of Oncologic Recurrence

AI models aimed at estimating post-LT oncologic risk have primarily focused on hepatocellular carcinoma (HCC) recurrence, integrating clinical, radiologic, and pathologic variables to support individualized risk assessment [33,50]. In a multicenter analysis, one ML approach reported a C-index of 0.75 (95%CI: 0.64–0.84) for predicting post-LT HCC recurrence, exceeding the performance of established clinical scoring systems [33]. Similar findings were observed for the Recurrent Liver Cancer Prediction Score (RELAPSE), developed using RSF and classification and regression tree methods. The RELAPSE showed a C-index of 0.81 in the development cohort and maintained comparable performance during external validation in a European population, with AUC-ROC values of 0.77 and 0.75 at 2 and 5 years, respectively [50].

#### 3.2.2. Mortality Prediction

Estimating post-transplant mortality risk is central to optimizing clinical pathways, guiding early interventions, and supporting individualized follow-up strategies. Eleven studies evaluated AI-based approaches for mortality prediction across diverse time horizons, recipient cohorts, and methodological frameworks [18,19,22,30,31,34,36,39,45,52,53]. For short-term postoperative outcomes, RF models demonstrated moderate discrimination, reporting an AUC-ROC of 0.771 for 30-day survival prediction [39]. Extending beyond the immediate postoperative period, RF models also performed favorably in predicting 1-, 3-, and 12-month mortality following deceased donor LT, achieving AUC-ROC values of 0.80, 0.85, and 0.81, respectively. These values exceeded those of commonly used prognostic tools such as MELD, donor MELD, and BAR, each of which consistently remained below an AUC-ROC of 0.70 [52]. More advanced DL architectures incorporating longitudinal and multimodal clinical data further improved predictive performance, with one model reporting an AUC-ROC of 0.92 for 1-year post-transplant survival [53]. Recent work has also explored expert-augmented ML frameworks. In this context, transformer-based models achieved robust long-term mortality prediction, with AUC-ROC values of 0.804 (95%CI: 0.773–0.835; *p* < 0.0001) for 1-year mortality and 0.733 (95%CI: 0.703–0.762; *p* < 0.0001) for 5-year mortality across heterogeneous causes of death, outperforming traditional logistic regression approaches [45].

#### 3.2.3. Infection Risk

Post-transplant infections remain a major source of morbidity in LT recipients; however, AI-based predictive models targeting infection-related outcomes are relatively sparse. Among the included studies, one investigation developed and validated multiple ML models to predict pressure injury in pediatric living donor LT recipients, an adverse event with infectious and wound-related implications [22]. Across the tested algorithms, the decision tree model showed the highest discriminative performance, reporting an AUC-ROC of 0.84 in the testing dataset [22]. Univariate analyses further identified prolonged operative duration (*p* = 0.001), intraoperative corticosteroid administration (*p* = 0.001), and absence of preoperative skin protection measures (*p* = 0.002) as significant risk factors for pressure injury development [22].

#### 3.2.4. Multimodal or Composite Outcome Prediction

AI models capable of integrating multimodal data streams and predicting composite clinical outcomes represent an emerging methodological direction in post-LT research. Sixteen studies evaluated such approaches, incorporating combinations of clinical, imaging, laboratory, and administrative data to address multiple outcome domains concurrently [16,17,21,25,29,32,37,38,40,41,42,47,49,51,54,56]. Owing to methodological heterogeneity, five studies were prioritized for detailed synthesis based on data integration strategies, direct model comparisons, and clinical relevance [16,17,25,38,54]. In the oncologic setting, a deep survival model integrating demographic, clinical, and imaging variables was developed to predict recurrence-free survival in LT recipients with HCC, reporting a C-index of 0.812 (±0.082) in testing and 0.839 (±0.001) in external validation, with significantly better recurrence stratification than the Milan criteria (*p* < 0.001) [25]. Beyond oncologic endpoints, multimodal approaches were also applied to major adverse cardiovascular events, with a DL model trained on large-scale claims data predicting events up to five years post-LT with an AUC-ROC of 0.763 (95%CI: 0.748–0.777) and an AUC-PR of 0.682 (95%CI: 0.661–0.711), outperforming conventional risk scores [17]. In pediatric LT, an RF classifier trained on the Studies of Pediatric Liver Transplantation (SPLIT) registry data predicted attainment of an “ideal composite outcome”, defined by sustained allograft function without immune or non-immune complications, with an accuracy of 0.71 (95%CI: 0.68–0.74), positive predictive value (PPV) of 0.83 (95%CI: 0.76–0.89), and negative predictive value (NPV) of 0.70 (95%CI: 0.68–0.71) [16]. A single-model DL study using pre-transplant clinical and laboratory variables reported excellent discrimination for early post-LT complications in hepatitis C-infected recipients (accuracy 100%, AUC-ROC 1.0, F2 score 1.0 in the validation cohort), although no head-to-head comparator was reported [54]. Multimodal models have also been applied to acute kidney injury, where gradient boosting techniques reported the highest performance among several ML comparators, with an AUC-ROC of 0.90 (95%CI: 0.86–0.93) and an accuracy of 84%, significantly exceeding logistic regression (AUC-ROC 0.61, 95%CI: 0.56–0.66, *p* < 0.001), RF (AUC-ROC 0.85, 95%CI: 0.81–0.89, *p* = 0.001), and decision tree models (AUC-ROC 0.86, 95%CI: 0.81–0.89, *p* = 0.033) [38].

### 3.3. Theme 2: Operational Outcomes

AI applications targeting operational aspects of post-LT care, such as real-time risk stratification, automated alerting, and clinical decision support, remain comparatively underrepresented in the literature, with three studies evaluating models designed to augment bedside workflows and facilitate earlier recognition of postoperative complications [57,58,59]. Table 2 summarizes the operational domains addressed, corresponding data modalities, and reported performance metrics.

One investigation developed an RF model integrating eight routinely collected clinical and laboratory variables to predict post-LT sepsis [58]. The RF model reported an AUC-ROC of 0.731 (95%CI: 0.649–0.802), outperforming the Sequential Organ Failure Assessment (SOFA) score, which achieved an AUC-ROC of 0.637 (95%CI: 0.551–0.692) [58]. To support clinical adoption, the model was deployed as an online calculator for bedside use [58]. In a related operational context, an AI-enabled early warning system leveraging electronic health records (EHRs), vital signs, and laboratory measurements was developed to predict postoperative pneumonia, achieving an AUC-ROC of 0.778 (95%CI: 0.720–0.836) compared with 0.647 (95%CI: 0.586–0.708) for the SOFA score (*p* < 0.001) [57]. Finally, an ensemble learning framework combining natural language processing of clinical notes with physiologic time-series and structured EHR data was applied to predict multiple adverse events, including rejection and infection [59]. This model reported an AUC-ROC of 0.79, supporting its potential role as an integrated decision-support component within post-LT workflows [59].

### 3.4. Theme 3: System-Level Outcomes

Unlike clinical and operational applications, system-level investigations explore how AI tools interact with the broader digital and organizational infrastructure surrounding post-LT care. These include considerations of interoperability, fairness, scalability, and deployment feasibility. Six studies examined such system-level dimensions [60,61,62,63,64,65]. Table 3 summarizes investigations focused on technical performance, cross-domain modeling, and integration challenges within post-transplant workflows.

Several studies emphasized multi-task modeling and equity considerations. One transformer-based framework simultaneously predicted five post-LT complications, malignancy, diabetes mellitus, rejection, infection, and cardiovascular events, while incorporating fairness constraints to reduce demographic performance disparities [65]. This model reported an AUC-ROC of 0.660 (95%CI: 0.650–0.670) for cardiovascular prediction, reflecting task-balanced rather than single-outcome optimization [65]. Another study developed a hybrid modeling approach integrating tree-based algorithms with deep neural networks (DNNs) to predict cause-specific mortality, reporting AUC-ROC values of 0.640 for rejection-related death and 0.646 for infection-related death [61]. In contrast to these model-centric architectures, one evaluation compared classical statistical methods with ML algorithms for donor–recipient matching, a system-level task with implications for organ allocation and resource use [60]. Logistic regression demonstrated superior performance for 5-year graft survival prediction, achieving an AUC-ROC of 0.654 and outperforming more complex ML techniques, emphasizing how limitations in data completeness and granularity may constrain the added value of advanced AI approaches in certain system-level contexts [60].

### 3.5. Additional Evidence Mapping

In addition to outcome-focused studies, several included publications synthesized AI applications across multiple transplant phases and clinical domains [11,66,67,68,69,70,71,72,73,74,75,76,77]. As shown in Table 4, these sources predominantly described supervised ML techniques (including RF, gradient boosting, ANN, and DNN), frequently discussed benchmarking against clinical scoring systems (such as MELD, BAR, and SOFT), and highlighted applications spanning outcomes including mortality, oncologic recurrence, sepsis, acute kidney injury, and rejection. Commonly reported methodological patterns included reliance on retrospective data, heterogeneous input features, and variable model performance reporting, with limited external or prospective validation across sources.

## 4. Discussion

This scoping review identifies a maturing yet uneven landscape of AI research in post-LT care. Most studies concentrated on supervised ML and DL approaches for clinical prediction tasks, where structured postoperative data and established prognostic benchmarks provide favorable conditions for algorithmic development. By contrast, relatively few studies extended AI applications toward bedside operational support or system-level integration, and even fewer addressed the steps required for real-world deployment. Taken together, these findings suggest that while technical capability for clinical risk modelling is advancing, the infrastructural, methodological, and implementation scaffolds necessary for clinical uptake remain comparatively underdeveloped.

Clinical prediction represents the most technically developed application domain for post-LT AI. Across graft survival, graft failure, rejection, fibrosis progression, oncologic recurrence, mortality, and composite adverse outcomes, supervised ML and DL models frequently demonstrated discrimination gains over traditional statistical tools, such as MELD, donor–MELD, BAR, and HCC recurrence indices [23,27,33,48,50,52]. These performance gains likely reflect the capacity of non-linear modelling to capture multidimensional postoperative trajectories shaped by perioperative factors, immune responses, and oncologic risk. Importantly, the predictive targets prioritized in this literature map directly onto high-stakes clinical decisions, including immunosuppression adjustment, surveillance intensity, and prognostic counselling, which likely explains why this segment has matured more rapidly than others.

By comparison, AI applications aimed at operational augmentation remain nascent. Only a small subset of studies developed early warning systems, automated triage algorithms, or risk-alerting models designed to function within bedside workflows [57,58,59]. These approaches demonstrated that integrating structured EHR data, physiologic time-series, or unstructured clinical notes is feasible and can outperform generic acuity scores, such as SOFA, for detecting postoperative pneumonia or sepsis risk. Such findings illustrate a logical extension of AI from static prediction toward workflow-embedded decision support. However, no studies assessed prospective use, clinician–algorithm interaction, usability, or alert burden, nor did any link algorithmic output to time-to-intervention or downstream clinical outcomes. Operational AI, therefore, provides technical signals of promise, but lacks evidence of compatibility with real-world transplant care processes.

System-level investigations were even more limited but offer insight into how AI could interface with the broader informational and organizational architecture of transplantation. Only six studies engaged with system-level questions [60,61,62,63,64,65], including multi-task learning frameworks modelling interdependent post-LT complications and hybrid architectures predicting cause-specific mortality. These designs move beyond single-endpoint prediction and reflect a more holistic representation of post-LT trajectories. Across these three domains, clinical, operational, and system-level, patterns emerged that shape the methodological and translational profile of current post-LT AI research.

**Figure 3:**
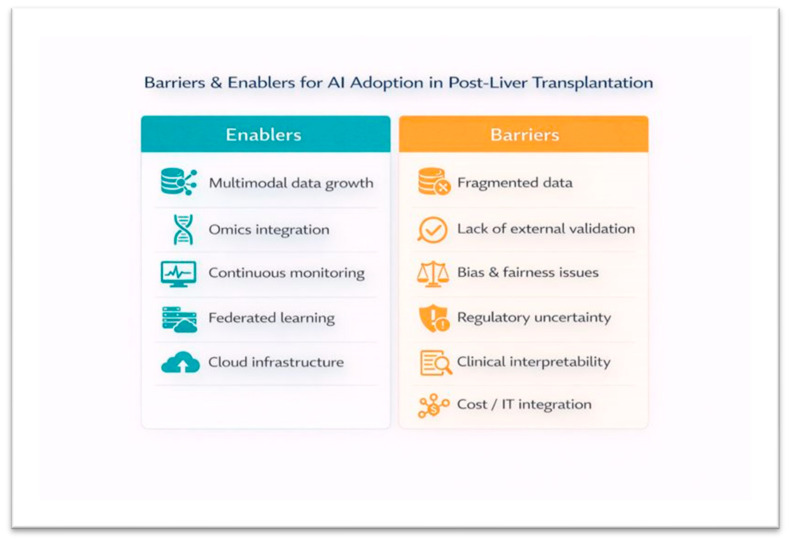
Barriers and enablers for AI adoption in post-liver transplantation care. A conceptual illustration of the principal enablers (**left**) and barriers (**right**) influencing clinical translation and deployment of AI systems in post-liver transplantation care. Enablers include multimodal data availability, omics integration, continuous monitoring, federated learning, and cloud-based infrastructure. Barriers include fragmented data ecosystems, limited external validation, bias and fairness considerations, regulatory uncertainty, lack of interpretability, and resource-intensive IT integration.

Across application domains, several methodological characteristics constrain interpretability, comparability, and translational potential. These methodological constraints parallel broader translational barriers and enablers for clinical implementation of AI in post-LT care, summarized in Figure 3. Most studies were retrospective and single-center, with heterogeneous variable sets and non-standardized outcome definitions [14,15,16,17,18,19,20,21,22,23,24,25,26,27,28,29,30,31,32,33,34,35,36,37,38,39,40,41,42,43,44,45,46,47,48,49,50,51,52,53,54,55,56,57,58,59,60,61,62,63,64,65]. Internal validation was common, external validation was uncommon, and prospective validation or real-world implementation was absent. While many included studies demonstrated promising discrimination, the majority represent proof-of-concept model development rather than clinically applicable AI. Most models were trained retrospectively and evaluated using internal cross-validation or split-sample testing, without evidence of prospective use, workflow integration, or impact on clinical decision-making. Only a limited subset of studies approached translational readiness through external validation in independent cohorts (e.g., multicenter validation of HCC recurrence and mortality prediction frameworks) [25,45,50,52]. A small number of operational studies also moved toward clinical usability by providing implementation-oriented interfaces such as online calculators or early warning systems [57,58]. However, even these deployment-adjacent investigations did not report clinician-in-the-loop evaluation, usability testing, alert burden, or downstream patient outcomes [57,58]. This distinction highlights a critical evidence gap: the current post-LT AI literature is rich in retrospective performance benchmarking, but remains sparse in clinically implemented, prospectively evaluated decision-support systems.

In parallel, reporting quality and performance characterization were frequently incomplete. Performance reporting overwhelmingly emphasized discrimination metrics (AUC-ROC or C-index), whereas calibration, net benefit, decision-curve analysis, and impact metrics were rarely reported. Yet calibration is essential for risk communication and thresholding, and decision-analytic measures are necessary to determine whether improved accuracy translates into meaningful clinical benefit. Deep architectures introduced additional challenges related to explainability, uncertainty, and missing-data handling, but few studies described feature attribution or other interpretability techniques. Collectively, these methodological patterns indicate that while technical performance in controlled settings is increasingly well characterized, the evidentiary foundation required for safe and effective deployment remains incomplete.

These observations have several implications for the trajectory of AI in post-LT care. Translational impact will require movement beyond model-centric development toward ecosystem-centric design. For clinical prediction models, priorities include harmonized outcome definitions, multicenter data linkage, and prospective evaluation capable of assessing real-world performance and clinical impact. For operational AI, progress will depend on EHR integration, clinician-in-the-loop interfaces, usability testing, and deployment studies measuring alert burden, time-to-intervention, and acceptance among transplant teams. For system-level AI, advancement will require collaborative data infrastructures, federated and privacy-preserving learning strategies, and governance frameworks enabling the secure use of transplant registries and claims data. Finally, alignment with emerging reporting and evaluation standards (TRIPOD-AI, PROBAST-AI, and DECIDE-AI) will be essential to support transparent and clinically meaningful translation.

Overall, the current evidence depicts a field transitioning from proof-of-concept model development toward questions of implementation, safety, and utility. The methodological foundations for accurate clinical prediction are increasingly established, yet the translational infrastructures necessary for real-world deployment remain limited. Future progress will require harmonizing methodological rigor with implementation science, human-factors engineering, and system-level integration. Only through such alignment can AI systems evolve from retrospective accuracy benchmarks into clinically integrated tools with the capacity to alter postoperative trajectories and improve outcomes for LT recipients.

## 5. Conclusions

AI research in post-LT care appears to be approaching an inflection point. Technical capacity to model clinically meaningful graft and patient trajectories is now well established, yet the translational pathways required to convert predictive performance into clinical utility remain underdeveloped. Progress will depend on the alignment of methodological rigor, data infrastructure, human-factors engineering, and implementation science. Whether AI becomes an analytical adjunct or an integrated component of LT care will depend not on incremental gains in discrimination, but on the development of the institutional, infrastructural, and regulatory scaffolds needed to support safe, equitable, and clinically meaningful deployment.

## Figures and Tables

**Table 1:** Studies reporting clinical outcomes predicted by artificial intelligence models in post-liver transplantation care.

Study Identification	Design and Methods	Population	AI Models Evaluated and Input Data	Comparative Framework	Implementation Status	Clinical Outcome Predicted and Model Performance	Post-LT Clinical Application
Abdelhameed A et al. [17] (United States, 2024)	Retrospective study using claims data from Optum Clinformatics (2007–2020); 5-fold cross-validation; external test set (20%).	*n* = 18,304 LT recipients.	BiGRU vs. baseline ML models; input: diagnoses, demographics, medications, procedures (3 years pre-LT).	Head-to-head comparison between BiGRU and traditional ML models.	Model development only (retrospective).	MACE at 30 days post-LT; BiGRU: AUC-ROC = 0.841 (95%CI, 0.822–0.862); AUC-PR = 0.578 (95%CI, 0.537–0.621).	Prediction of MACE.
Andishgar A et al. [18] (Iran, 2025)	Retrospective study using clinical data from a single transplant center (2018–2023); 5-fold cross-validation; random oversampling; hyperparameter tuning.	*n* = 1799 LT recipients.	Seven ML survival models (LASSO, Ridge, RSF, E-NET, GBS, C-GBS, FS-SVM); input: 40 clinical predictors (e.g., graft type, BMI, AST, creatinine, tacrolimus use).	Head-to-head comparison of survival models with three feature selection techniques: Cox-P, RSF-based selection, and LASSO.	Model development only (retrospective).	Biliary complications; RSF + Ridge: C-index = 0.699; mortality; RSF + RSF: C-index = 0.784.	Prediction of biliary complications and mortality.
Andres A et al. [19] (Canada, 2018)	Retrospective study using data from the SRTR (2002–2013); D-calibration and Hosmer–Lemeshow calibration.	*n* = 2769 adult LT recipients with PSC.	PSSP algorithm vs. Cox regression; input: patient-level clinical features.	Head-to-head comparison of calibrated survival predictions using D-calibration and single-time calibration tests.	Model development only (retrospective).	Post-LT survival in PSC; PSSP: D-calibration: *p* = 1.0; Hosmer–Lemeshow: *p* = 0.802 (0.25-year), *p* = 0.502 (1-year), *p* = 0.173 (5-year), *p* = 0.169 (10-year); Cox model: failed calibration at 10 years (*p* = 0.027).	Prediction of long-term survival in PSC.
Azhie A et al. [20] (Canada, 2024)	Retrospective longitudinal study using clinical and biopsy data from a single Canadian transplant center (1987–2019); subgroup validation using transient elastography.	*n* = 1893 adult LT recipients with ≥1 post-LT liver biopsy.	Weighted LSTM model vs. logistic regression, decision tree, AdaBoost, GBDT, XGBoost, and random forest; input: longitudinal clinical and laboratory data.	Head-to-head comparison of model performance for prediction of biopsy-confirmed fibrosis stage.	Model development only (retrospective).	Significant graft fibrosis (≥F2) post-LT; weighted LSTM: AUC-ROC = 0.798 (95%CI, 0.790–0.810); sensitivity = 0.83; specificity = 0.81.	Prediction of significant graft fibrosis (≥F2).
Bezjak M et al. [21] (Croatia, 2023)	Retrospective study using clinical data from a single transplant center (2013–2019); 5-fold cross-validation; cross-validated hyperparameter tuning; holdout test set.	*n* = 170 adult LT recipients with HCC.	RSF, survival SVM, survival gradient boosting, and Coxnet; input: 30 donor, recipient, and tumor-specific clinical parameters.	Head-to-head comparison of survival models using concordance index.	Model development only (retrospective).	Recurrence-free survival post-LT for HCC; RSF: C-index = 0.72 (highest among models).	Prediction of recurrence-free survival, overall survival, graft survival, and HCC recurrence.
Bhat V et al. [22] (United States, 2018)	Retrospective study using data from the SRTR (1987–2016).	*n* = 60,054 adult LT recipients.	Random forest, ANN, gradient boosting, and SVM; input: donor, recipient, and transplant characteristics.	Head-to-head comparison of models for prediction of NODAT; survival analysis comparing outcomes by DM status.	Model development only (retrospective).	Significant predictors of NODAT included age, sex, obesity, and sirolimus use; patients with NODAT had lower 10-year survival than those without DM (63.0% vs. 74.9%, *p* < 0.001).	Prediction of NODAT and long-term survival.
Briceño J et al. [23] (Spain, 2014)	Retrospective study using clinical data from seven Spanish LT centers (2005–2009).	*n* = 529 adult LT recipients.	ANN vs. logistic regression; input: 23 donor and recipient clinical variables.	Head-to-head comparison of ANN and logistic regression for graft survival prediction.	Model development only (retrospective).	3-month graft survival post-LT; ANN: AUC-ROC = 0.76; logistic regression: AUC-ROC = 0.69; *p* < 0.001.	
Calleja R et al. [24] (Spain, 2025)	Retrospective study using clinical data from 25 Spanish transplant centers.	*n* = 420 adult cDCD-NRP LT recipients.	Logistic regression, ridge classifier, SVM, MLP, and random forest; input: 14 donor–recipient variables including age, MELD, CIT, and WIT.	Head-to-head comparison of five models to predict 3- and 12-month graft survival.	Model development only (retrospective).	3-month and 12-month graft survival post-LT cDCD-NRP; ridge classifier: AUC-ROC = 0.78 (3 months); AUC-ROC = 0.72 (12 months).	Prediction of graft survival in cDCD-NRP recipients.
Cao S et al. [25] (China, 2025)	Retrospective study using clinical data from three transplant centers in China (2015–2021); external validation at two independent centers.	*n* = 466 adult LT recipients with HCC.	DeepSurv (pre- and postoperative models) vs. logistic regression, stacking, SVM, and random forest; input: clinical and clinicopathologic variables.	Head-to-head comparison of survival models and clinical criteria (Milan, UCSF, RETREAT) for recurrence prediction.	Externally validated (independent cohort).	HCC recurrence post-LT; post-DeepSurv model: C-index = 0.835 (training); 0.812 (testing); 0.839 and 0.831 (external validation).	Prediction of HCC recurrence.
Chen X et al. [26] (China, 2024)	Retrospective study using clinical data from a single pediatric transplant center (2021–2022); internal validation performed.	*n* = 438 pediatric LDLT recipients.	Decision tree, random forest, gradient boosting decision tree, and XGBoost; input: 10 perioperative and preoperative clinical variables including operation time, corticosteroid use, and skin condition.	Head-to-head comparison of four ML models for prediction of pressure injuries.	Model development only (retrospective).	Pressure injury post-LT in children; decision tree: AUC-ROC = 0.841; accuracy = 0.848; sensitivity = 0.769; specificity = 0.857.	Prediction of pressure injury.
Cooper JP et al. [27] (United States, 2022)	Retrospective study using clinical data from a single transplant center (1996–2019); internal validation and additional validation in a separate cohort (2019–2020).	*n* = 1938 adult LT recipients.	Logistic regression, C5.0, heterogeneous ensemble, generalized gradient boosting machine, and three other ML models; input: donor and recipient clinical variables.	Head-to-head comparison of seven ML models for prediction of GVHD.	Model development only (retrospective).	Acute GVHD post-LT; all models: AUC-ROC = 0.83–0.86 (test set); AUC-ROC = 0.93–0.96 (validation set).	Prediction of GVHD.
Cruz-Ramírez M et al. [28] (Spain, 2013)	Retrospective study using clinical data from eleven Spanish LT centers (2007–2008); 3-month post-transplant follow-up; model training with MPENSGA2 algorithm.	*n* = 248 adult LT recipients.	Evolutionary multi-objective radial basis function ANN trained using MPENSGA2 algorithm; input: donor, recipient, and transplant variables.	Head-to-head comparison of Pareto-optimized ANN selected for accuracy and sensitivity.	Model development only (retrospective).	3-month graft survival post-LT; optimized ANN: AUC-ROC = 0.85; correct classification rate = 85.9%; minimum sensitivity = 85.5%.	Prediction of 3-month graft survival.
Ding Z et al. [29] (China, 2025)	Retrospective study using clinical data from a single transplant center (2014–2022); internal validation and external validation using the MIMIC-IV dataset.	*n* = 1370 adult LT recipients.	Logistic regression, SVM, random forest, LightGBM, and XGBoost; input: 49 perioperative variables including age, bilirubin, and anesthesia-related factors.	Head-to-head comparison of five models for PND prediction across internal and external cohorts.	Externally validated (independent cohort).	PND post-LT; logistic regression: AUC-ROC = 0.799 (internal); AUC-ROC = 0.826 (temporal external); AUC-ROC = 0.720 (MIMIC-IV).	Prediction of PND.
Fatemi Y et al. [30] (United States, 2024)	Retrospective study using SRTR (UNOS) transplant database (1987–2022); model development with 5-fold cross-validation and 100 bootstrap samples; feature selection via RFE and SFM; SHAP used for model interpretability.	*n* = 10,871 adult NASH LT recipients.	XGBoost, random forest, decision tree, SVM, KNN, and naïve Bayes; input: 92 pre-LT donor and recipient features.	Head-to-head comparison of six ML models across eight feature selection strategies for cardiovascular mortality prediction.	Model development only (retrospective).	Cardiovascular death post-LT in NASH; XGBoost with RFE-random forest: AUC-ROC = 0.86; accuracy = 69.1%; SHAP applied to rank predictor importance and improve interpretability.	Prediction of cardiovascular death in NASH recipients.
Ge J et al. [31] (United States, 2023)	Retrospective cohort study using SRTR data (2010–2020) from 129 centers; 5-fold cross-validation; model calibration and SHAP-based interpretability assessed.	*n* = 49,121 adult LT recipients.	DNN, logistic regression, random forest; input: 37 pre-LT clinical variables (e.g., comorbidities, MELD, albumin).	Head-to-head comparison of DNN, logistic regression, and random forest for 90-day and 1-year mortality and 90-day readmission prediction.	Model development only (retrospective).	90-day and 1-year mortality; 90-day readmission post-LT; DNN: AUC-ROC = 0.737 (1-year mortality); AUC-ROC = 0.727 (90-day mortality); AUC-ROC = 0.651 (readmission).	Prediction of mortality and hospital readmission.
He T et al. [32] (United States, 2021)	Retrospective study using clinical data from a single transplant center (2008–2019); 5-fold cross-validation; model interpretability assessed.	*n* = 137 LT recipients with HCC.	i-RAPIT DL model; input: postoperative CT images and clinical variables (e.g., Milan criteria, tumor count).	Head-to-head comparison of i-RAPIT with logistic regression and Milan/AFP models for HCC recurrence prediction.	Model development only (retrospective).	HCC recurrence post-LT; i-RAPIT: AUC-ROC = 0.89; F1 score = 0.90; accuracy = 91.3%; precision = 0.90; recall = 0.90.	Prediction of HCC recurrence.
Ivanics T et al. [33] (Canada, 2022)	Retrospective study using clinical, imaging, and treatment data from LT recipients listed 2000–2016; cross-validation and held-out test set.	*n* = 739 LT recipients with HCC.	CoxNet, survival random forest, survival SVM, DeepSurv; input: serial imaging, AFP, locoregional therapies, treatment response.	Head-to-head comparison of survival models using concordance index; CoxNet validated against AFP, MORAL, and HALT-HCC scores.	Model development only (retrospective).	HCC recurrence post-LT; CoxNet: C-index = 0.75 (95%CI, 0.64–0.84); AFP and MORAL scores: C-index = 0.64; HALT-HCC: C-index = 0.72 (not significantly outperformed).	Prediction of HCC recurrence.
Jain V et al. [34] (United States, 2021)	Retrospective cohort study using clinical data from a single transplant center (2008–2019); 5-fold cross-validation.	*n* = 1459 LT recipients.	Logistic regression, LASSO, random forest, SVM, and XGBoost; input: pre-LT clinical variables including age, DM, creatinine, right ventricular systolic pressure, and left ventricular ejection fraction.	Head-to-head comparison of five ML models using AUC-ROC and Harrell’s C statistic.	Model development only (retrospective).	MACE: XGBoost: AUC-ROC = 0.71 (95%CI, 0.63–0.79); all-cause mortality: Harrell’s C = 0.64 (95%CI, 0.57–0.73); cardiovascular mortality: AUC-ROC = 0.72 (95%CI, 0.59–0.85).	Prediction of MACE, all-cause mortality, and cardiovascular mortality.
Kantidakis G et al. [35] (United Kingdom, 2020)	Retrospective study using clinical data from the SRTR (2005–2015); 10-year follow-up; internal validation performed.	*n* = 529 LT recipients.	Random survival forest, ANN, Cox proportional hazards; input: 23 donor and recipient variables (e.g., age, MELD, diagnosis, cold ischemia time).	Head-to-head comparison of ML models with Cox regression for graft survival prediction.	Model development only (retrospective).	Graft survival post-LT; RSF: C-index = 0.68; ANN: C-index = 0.66; Cox: C-index = 0.67.	Prediction of graft survival.
Kazemi A et al. [36] (Iran, 2019)	Retrospective study using clinical data from a single transplant center (2011–2014); internal validation performed.	*n* = 529 LT recipients.	SVM, MLP, Bayesian network, C5.0, KNN; input: 23 donor and recipient clinical variables (e.g., age, MELD, diagnosis, CIT).	Head-to-head comparison of five ML models and Cox regression for 6-month survival prediction.	Model development only (retrospective).	6-month mortality post-LT; MLP: AUC-ROC = 0.77; accuracy = 80.0%; F1 score = 0.78; sensitivity = 0.78; specificity = 0.81; precision = 0.78.	Prediction of short-term mortality.
Ko SH et al. [37] (South Korea, 2024)	Retrospective study using clinical data from a single transplant center (2015–2018); internal validation performed.	*n* = 466 LT recipients with HCC.	TabNet; input: 35 pre- and post-LT clinical and pathologic variables.	Head-to-head comparison of TabNet vs. Milan, UCSF, and RETREAT criteria for HCC recurrence prediction.	Model development only (retrospective).	HCC recurrence post-LT; TabNet: AUC-ROC = 0.825; C-index = 0.796; accuracy = 0.841; NRI = 36.9% vs. Milan criteria; HR = 4.74 (95%CI, 2.72–8.24) for high-risk group.	Prediction of HCC recurrence.
Lee HC et al. [38] (South Korea, 2018)	Retrospective study using clinical data from a single transplant center (2008–2016); internal validation performed.	*n* = 1370 LT recipients.	Gradient boosting, random forest, decision tree, SVM, ANN; input: 49 intraoperative variables (e.g., anesthesia records, hemodynamics, laboratory).	Head-to-head comparison of five ML models for prediction of AKI post-LT.	Model development only (retrospective).	AKI post-LT; gradient boosting: AUC-ROC = 0.86; accuracy = 81.3%; *p* < 0.001 vs. logistic regression.	Prediction of AKI.
Liu CL et al. [39] (Taiwan, 2020)	Retrospective study using clinical data from a single transplant center (2004–2013); 10-fold cross-validation; temporal validation using 2013 cohort.	*n* = 538 LT recipients.	Random forest, XGBoost, logistic regression, decision tree; input: 8 preoperative features (e.g., BMI, INR, lymphocyte, WBC, sodium).	Head-to-head comparison of four ML models for 30-day survival prediction.	Model development only (retrospective).	30-day survival post-LT; random forest: AUC-ROC = 0.771 (temporal); specificity = 0.815; sensitivity = 0.5; C-index = 0.85 (derivation set).	Prediction of 30-day survival.
Liu LP et al. [40] (China, 2021)	Retrospective study using clinical data from three transplant centers (2014–2019); 5-fold cross-validation; prospective validation performed.	*n* = 1193 LT recipients.	XGBoost, GBDT, random forest, AdaBoost, SVM, MLP, KNN, naïve Bayes, logistic regression; input: 24 pre-LT variables (e.g., age, hemoglobin, APTT, direct bilirubin).	Head-to-head comparison of nine models for RBC transfusion prediction.	Not reported.	RBC transfusion during or after LT; XGBoost: AUC-ROC = 0.813; sensitivity = 66.4%; specificity = 85.0%.	Prediction of RBC transfusion.
Liu Z et al. [41] (China, 2022)	Retrospective study using clinical data from a single transplant center (2015–2019); external validation in TCGA cohort.	*n* = 144 LT recipients with HCC.	MobileNetV2-based classifier; input: histological tiles with nuclear architectural features extracted by U-net.	Head-to-head comparison of model vs. clinical variables (e.g., AJCC stage, AFP, tumor number).	Externally validated (independent cohort).	HCC recurrence post-LT; MobileNetV2-based classifier: HR = 3.44 (95%CI, 2.01–5.87); model showed higher AUC-ROC and net reclassification improvement.	Prediction of HCC recurrence.
Loosen SH et al. [42] (Germany, 2023)	Retrospective cohort study using the Disease Analyzer database (2005–2020); internal validation performed.	*n* = 216 LT recipients.	Random forest, logistic regression, and XGBoost; input: diagnosis and prescription data within 12 months post-LT.	Head-to-head comparison of three ML models for NODAT prediction.	Model development only (retrospective).	NODAT within 12 months post-LT; random forest: AUC-ROC = 0.775; accuracy = 79.5%; sensitivity = 75.0%; specificity = 80.0%.	Prediction of NODAT.
Melvin DG et al. [43] (United Kingdom, 2000)	Retrospective study using clinical data from a single transplant center; biochemical and hematological data collected for up to 100 days post-LT; 100-fold pseudorandom training–validation splits.	*n* = 80 LT recipients.	MLP, logistic regression, LDA; input: liver function tests (ALT, ALP, GST, bilirubin), their gradients, and postoperative day.	Head-to-head comparison of nonlinear (MLP) vs. linear models (LDA, logistic regression) for rejection prediction.	Model development only (retrospective).	Biopsy-confirmed acute rejection; MLP: AUC-ROC up to 0.85 (validation); improved early detection vs. linear models.	Prediction of acute rejection.
Ningappa M et al. [44] (United States, 2022)	Retrospective study using transcriptomic data from a single pediatric transplant center; sampling included pre-LT, 0–90 days post-LT, and 2–5 years post-LT; 10-fold cross-validation; model training with LASSO regularization.	*n* = 185 pediatric LT recipients; *n* = 75 pre-LT, *n* = 55 early post-LT, and *n* = 55 late post-LT.	LASSO-based network model; input: blood transcriptomics overlaid on protein–protein interaction network.	Head-to-head comparison of network-based vs. gene-based and pathway-based models; permutation testing applied.	Model development only (retrospective).	Acute cellular rejection post-LT; LASSO model: AUC-ROC = 0.70 (pre-LT), = 0.65 (early and late post-LT); predictive gene modules identified.	Prediction of acute rejection.
Nitski O et al. [45] (Canada and United States, 2021)	Retrospective study using SRTR (United States, 2003–2014) and UHN (Canada, 1986–2014) datasets; SRTR: internal validation with 10% holdout; UHN: 5-fold cross-validation.	SRTR: *n* = 42,146; UHN: *n* = 3269.	Transformer, temporal convolutional network, recurrent neural network, MLP, logistic regression; input: pre- and post-LT clinical variables.	Head-to-head comparison of five models; SRTR: internal validation; UHN: external validation with transfer learning.	Externally validated (independent cohort).	Cause-specific mortality post-LT (cardiovascular, infection, cancer, graft failure); Transformer: AUC-ROC = 0.804 (1-year), 0.733 (5-year) in SRTR; 0.807 (1-year), 0.722 (5-year) in UHN; best 1-year AUC-ROC for graft failure = 0.859 (SRTR); best 5-year AUC-ROC for cancer = 0.764 (UHN).	Prediction of cause-specific mortality (cardiovascular, infection, cancer, graft failure).
Piscaglia F et al. [46] (Italy, 2006)	Retrospective study using clinical and laboratory data from a single transplant center (1998–2004); internal validation performed.	*n* = 188 adult LT recipients with recurrent HCV.	ANN vs. logistic regression; input: 7 clinical and laboratory variables (cholesterol, AST, ALP, albumin, sodium, platelet count, prothrombin time); *n* = 510 biopsies.	Head-to-head comparison of ANN and logistic regression for fibrosis prediction.	Model development only (retrospective).	Significant graft fibrosis (≥F3); ANN: AUC-ROC = 0.93 (95%CI, 0.86–0.97); sensitivity = 100%; specificity = 79.5%; NPV = 100%; PPV = 60.5%; *p* = 0.045 vs. logistic regression.	Prediction of significant graft fibrosis (≥F3).
Qu WF et al. [47] (China, 2023)	Retrospective study using clinical and pathological image data from a single transplant center (2005–2019); 10-fold cross-validation.	*n* = 380 adult LT recipients with HCC.	ResNet-50 and modified DeepSurv; input: hematoxylin and eosin-stained whole slide images classified into six tissue types (tumor, fibrous tissue, immune cells, portal area, hemorrhagic/necrotic tissue, normal liver).	Head-to-head comparison with Milan, UCSF, TNM, BCLC, ERASL-post, and RETREAT criteria for HCC recurrence prediction.	Model development only (retrospective).	HCC recurrence post-LT; DeepSurv model: C-index = 0.827 (training), 0.794 (validation); AUC-ROC at 1/2/5 years = 0.810/0.805/0.781 (training), 0.779/0.828/0.814 (validation).	Prediction of HCC recurrence.
Raji CG et al. [48] (India, 2025)	Retrospective cohort study using the UNOS dataset (2001–2023); 10-fold cross-validation; model training; survival analysis over 23 years.	*n* = 141,889 adult LT recipients; recorded deceased donor dataset: *n* = 135,709; recorded living donor dataset: *n* = 6180.	Deeplearning4j MLP vs. actual graft survival; input: 23 top-ranked attributes from UNOS dataset including donor, recipient, and transplant variables.	Head-to-head comparison of predicted vs. actual graft survival across recorded deceased and recorded living donor datasets.	Model development only (retrospective).	Graft survival post-LT; recorded living donor: accuracy = 99.91%, sensitivity = 99.9, specificity = 99.9; recorded deceased donor: accuracy = 99.86%, sensitivity = 99.7, specificity = 99.7.	Prediction of graft survival in living and deceased donor LT recipients.
Robertson H et al. [14] (Australia, 2024)	Retrospective meta-analysis using transcriptomics data from 150 datasets (pre-September 2022); >12,000 samples across kidney, liver, heart, and lung transplants; transfer learning models developed and validated; additional validation using AUSCAD cohort.	*n* = 12,970 transplant samples (kidney: 8853; liver: 1216; heart: 1160; lung: 1241); AUSCAD: *n* = 70 kidney/ kidney-pancreas recipients.	TOP models; input: whole blood and biopsy transcriptomic data.	Pan-organ vs. organ-specific models; external validation with AUSCAD cohort.	Externally validated (independent cohort).	Pan-organ model outperformed organ-specific and clinical models in predicting acute rejection (AUC-ROC = 0.81 vs. 0.70 vs. 0.58); fibrosis: AUC-ROC = 0.81; DGF: AUC-ROC = 0.89.	Prediction of acute rejection, delayed graft function, and fibrosis.
Rodriguez-Luna H et al. [49] (United States, 2005)	Retrospective study using clinical and tissue genotyping data from a single transplant center (1999–2002); follow-up = 18–72 months.	*n* = 19 adult LT recipients with HCC.	ANN + TM-GTP; input: histopathologic features and microsatellite mutation profiles (1p, 3p, 5q, 9p, 17p, 18q).	Head-to-head comparison of ANN alone vs. combined ANN + TM-GTP for HCC recurrence prediction.	Model development only (retrospective).	HCC recurrence post-LT; combined model: discrimination power = 89.5% (17/19); sensitivity = 100%; specificity = 100%; AUC-ROC = 1.00 (blind validation).	Prediction of HCC recurrence.
Tran BV et al. [50] (United States, 2023)	Retrospective study using clinical, radiologic, and pathologic data from UMHTC; external validation using EHCLT; competing risk regression and ML models (RSF, CART); external validation at 2 and 5 years.	*n* = 4981 LT recipients with HCC (United States); *n* = 1160 (Europe).	Random survival forest, classification and regression tree; input: AFP, neutrophil-lymphocyte ratio, tumor diameter, vascular invasion, differentiation, and other clinico-pathologic variables.	Head-to-head comparison of multivariable Fine-Gray model and ML-based risk scores (e.g., RSF) for recurrence prediction.	Externally validated (independent cohort).	HCC recurrence post-LT; Fine–Gray model: C-index = 0.78; RSF: C-index = 0.81; external validation AUC-ROC = 0.77 (2-year), 0.75 (5-year).	Prediction of HCC recurrence.
Tusch G et al. [15] (Germany, 1999)	Prospective longitudinal study using clinical data from a single transplant center (1974–1994); follow-up until July 1997; sequential decision modeling across three clinical time points; error constraints applied; comparison of linear discriminant and neural models.	*n* = 314 adult LT recipients with HCC.	LDA and adaptive MLP; input: 10 selected pre-, peri-, and postoperative clinical variables; missing values imputed.	Head-to-head comparison of LDA vs. adaptive MLP using constrained sequential decision framework.	Not reported.	High-risk patient classification (survival <2 years); adaptive MLP: sensitivity = 100%, specificity = 100%, AUC-ROC = 1.00 (blind validation); LDA performed comparably but with lower robustness.	Prediction of short-term mortality.
Wadhwani SI et al. [16] (Canada and United States, 2021)	Prospective cohort study using SPLIT registry data (2002–2006); 69 predictor variables evaluated at 1-year post-LT; random forests analysis.	*n* = 887 pediatric LT recipients.	Random forest; input: 69 demographic, perioperative, and 1-year post-LT clinical variables.	No explicit baseline comparator; variable importance derived from random forests.	Not reported.	Ideal outcome at 3 years; random forest; accuracy = 0.71 (95%CI, 0.68–0.74); PPV = 0.83; NPV = 0.70.	Prediction of ideal outcome at 3 years.
Xie H et al. [51] (China, 2024)	Retrospective study using clinical, radiomics, and pathological data from a single transplant center (2013–2018); 10-fold cross-validation.	*n* = 139 adult LT recipients with HCC.	Clinical model (AFP, ALP), pathological model (Ki-67, tumor number), radiomics model (Rad-score), nomogram model (NM) integrating all features; input: preoperative CT images and clinical/pathological data.	Head-to-head comparison of clinical, pathological, radiomics, and nomogram models for disease-free survival prediction.	Model development only (retrospective).	Disease-free survival post-LT in HCC patients; nomogram model: AUC-ROC = 0.882 (1-year), 0.867 (2-year), 0.882 (3-year) in training; AUC-ROC = 0.854 (1-year), 0.849 (2-year), 0.801 (3-year) in validation; C-index = 0.817 (training), 0.760 (validation).	Prediction of disease-free survival in HCC patients.
Yasodhara A et al. [52] (Canada and United States, 2021)	Retrospective study using SRTR dataset (1987–2019); external validation using UHN dataset (1989–2014); 5-fold cross-validation.	*n* = 18,058 adult LT recipients from SRTR; *n* = 1290 from UHN.	Cox proportional hazards, gradient boosting survival; input: pre- and post-LT clinical variables (e.g., DM status, hypertension, serum creatinine, BMI, immunosuppression).	Head-to-head comparison of CoxPH and GBS models across no DM, pre-DM, and NODAT groups using AUC-ROC, AUPR, and C-index in SRTR and UHN datasets.	Externally validated (independent cohort).	Long-term mortality post-LT; AUC-ROC = 0.60–0.72; AUPR = 0.15–0.37; C-index = 0.58–0.70 (SRTR and UHN).	Prediction of long-term mortality in LT recipients with and without DM.
Yu YD et al. [53] (South Korea, 2022)	Retrospective study using clinical data from the Korean Organ Transplant Registry (2014–2019); model training and validation repeated 25 times.	*n* = 785 adult LT recipients.	Random forest, ANN, decision tree, naïve Bayes, SVM; input: donor and recipient demographic and clinical features.	Head-to-head comparison of five ML models with Cox regression, MELD, donor MELD, and BAR scores.	Model development only (retrospective).	1-/3-/12-month survival; random forest: AUC-ROC = 0.80/0.85/0.81; outperforming MELD, donor MELD, and BAR (all AUC-ROC < 0.70).	Prediction of short- and medium-term survival.
Zabara ML et al. [54] (Romania, 2023)	Retrospective study using clinical and laboratory data from two transplant centers (2000–2017); model development using clinical data from 80 LT recipients; internal validation performed using 10 additional cases.	*n* = 90 LT recipients with hepatitis C.	Deep learning model (sequential network with dense layers); input: 14 pre-LT clinical and laboratory parameters.	N/A	Model development only (retrospective).	Prediction of short-term postoperative complications (≤30 days); accuracy = 100% (validation); AUC-ROC = 1.0; F2 score = 1.0.	Prediction of postoperative complications in hepatitis C-positive recipients.
Zalba Etayo B et al. [55] (Spain, 2023)	Retrospective study using clinical data from a single transplant center (2010–2021); internal validation performed.	*n* = 596 adult LT recipients.	ANN (MLP); input: donor age, donor type (donation after brain death), recipient age, cause of liver disease, transplant year, hepatitis C infection, cardiovascular risk factors, antithrombotic treatment, immunosuppression, portal vein thrombosis, and HCC.	Model performance evaluated against historical data; variable importance assessed using information value.	Model development only (retrospective).	Graft survival within 1 year post-LT; ANN: C-statistic = 0.745 (95%CI, 0.692–0.798); key predictors included recipient age, donor age, antithrombotic treatment, immunosuppression, and portal thrombosis.	Prediction of 1-year graft survival.
Zhang Y et al. [56] (China, 2021)	Retrospective study using clinical data from a single transplant center (2015–2019); 5-fold cross-validation; 1000 bootstrap iterations for internal validation; external temporal validation (2019–2021).	*n* = 780 adult LT recipients.	Logistic regression, SVM, random forest, AdaBoost, gradient boosting machine; input: 14 selected preoperative and intraoperative variables.	Head-to-head comparison of five ML models and Kalisvaart’s AKI prediction score for post-LT AKI prediction.	Externally validated (independent cohort).	AKI post-LT; GBM: AUC-ROC = 0.76 (95%CI, 0.70–0.82; internal), 0.75 (95%CI, 0.67–0.81; external); F1 score = 0.73 (95%CI, 0.66–0.78); sensitivity = 0.74 (95%CI, 0.66–0.80); specificity = 0.65 (95%CI, 0.55–0.73).	Prediction of AKI.

**Table 2:** Studies reporting operational outcomes of artificial intelligence models in post-liver transplantation care.

Study Identification	Design and Methods	Population	AI Models Evaluated and Input Data	Comparative Framework	Implementation Status	Operational Performance and Decision-Support Impact	Post-LT Clinical Application
Chen C et al. [57] (China, 2021)	Retrospective study using clinical data from a single transplant center (2015–2019); random train/test split.	*n* = 591 adult LT recipients.	Logistic regression, SVM, random forest, AdaBoost, XGBoost, and gradient boosting machine; input: 14 perioperative clinical and laboratory variables.	Head-to-head comparison of six ML models for pneumonia prediction.	Model development only (retrospective).	Postoperative pneumonia prediction post-LT; XGBoost: AUC-ROC = 0.794 (95%CI, 0.735–0.84); sensitivity = 61.8%; specificity = 81.5%; pneumonia associated with increased hospitalization and lower 3-year survival (*p* < 0.05).	Prediction of postoperative pneumonia to support early risk stratification and intervention.
Chen C et al. [58] (China, 2023)	Retrospective study using clinical data from a single transplant center (2015–2020); external validation at the same center (2020–2021).	*n* = 677 adult LT recipients.	Random forest classifier vs. six other ML models; input: 8 pre- and intraoperative variables (e.g., blood loss, anesthesia time, and preoperative total bilirubin).	Head-to-head comparison of seven ML models for prediction of postoperative sepsis.	Deployed as calculator/tool.	Sepsis prediction within 7 days post-LT; random forest: AUC-ROC = 0.731 (internal), AUC-ROC = 0.755 (external); sensitivity = 62.1%; specificity = 76.1%; sepsis associated with increased complications, ICU/hospital stay, cost, and mortality at 30 and 90 days ( < 0.05).	Prediction of postoperative sepsis to support early identification and timely intervention.
Kamaleswaran R et al. [59] (United States, 2021)	Retrospective study using clinical data from a single transplant center (2017–2020); internal validation; 12 h sliding window analysis.	*n* = 298 LT recipients.	XGBoost; input: continuous physiological signals (heart rate, respiratory rate, blood pressure, and SpO_2_).	Head-to-head comparison of XGBoost vs. baseline logistic regression for early sepsis prediction.	Model development only (retrospective).	Early sepsis detection post-LT; XGBoost: AUC-ROC = 0.87; sensitivity = 85.3%; specificity = 77.1%; PPV = 83.1%; generated alerts up to 12 h before clinical recognition.	Prediction of post-LT sepsis to enable early intervention and improve monitoring.

**Table 3:** Studies reporting system-level outcomes of artificial intelligence models in post-liver transplantation care.

Study Identification	Design and Methods	Population	AI Models Evaluated and Input Data	Comparative Framework	Implementation Status	Integration And System-Level Performance	Post-LT Clinical Application
Ding S et al. [60] (United States, 2023)	Retrospective study using the STAR dataset; 5-fold cross-validation.	*n* = 160,360 LT recipients.	Fair-ML model vs. logistic regression, random forest, and GBDT; input: 80 recipient and donor features.	Head-to-head comparison of ML models with and without two-step fairness debiasing.	Model development only (retrospective).	Graft failure post-LT; Fair-ML model: AUC-ROC = 0.792; DPD = 0.597; EOD = 0.662.	Prediction of graft failure to inform fair organ assignment.
Ding S et al. [61] (United States, 2024)	Retrospective study using clinical data from the STAR dataset (2002–2021); 5-fold cross-validation.	*n* = 160,460 adult LT recipients.	CoD-MTL vs. baseline tree-based models; input: 80 recipient and donor variables.	Head-to-head comparison of CoD-MTL with baseline models for multi-label classification.	Model development only (retrospective).	Post-transplant cause of death (rejection and infection); CoD-MTL: AUC-ROC = 0.83 (average); AUC-PR = 0.38; calibration slope = 1.03; intercept = −0.02; DPD = 0.61; EOD = 0.53.	Prediction of multiple causes of death using multi-task learning.
Dorado-Moreno M et al. [62] (Spain and United Kingdom, 2017)	Retrospective study using transplant data from 7 Spanish hospitals (2007–2008) and King’s College Hospital, United Kingdom (2002–2010); 12-month follow-up; model evaluated using 5-fold cross-validation.	*n* = 248 LT donor–recipient pairs	Evolutionary ordinal ANN, SVM, random forests, and gradient boosted trees; input: donor, recipient, and surgical features.	Head-to-head comparison of ordinal classifiers for graft viability prediction.	Model development only (retrospective).	Graft survival post-LT; evolutionary ordinal ANN: accuracy = 86.3%; GMS = 81.0%; AMAE = 0.29.	Prediction of graft viability to support organ allocation decisions.
Guijo-Rubio D et al. [63] (Spain, 2021)	Retrospective study using national LT database from 24 Spanish hospitals (from 2004); 5-fold cross-validation; 3-month to 5-year follow-up endpoints.	*n* = 2914 LT donor–recipient pairs.	Logistic regression, MLP, random forest, SVM, KNN, gradient boosting; input: donor and recipient clinical variables.	Head-to-head comparison of six models across 3-month to 5-year graft survival prediction tasks.	Model development only (retrospective).	Graft survival post-LT; gradient boosting: AUC-ROC = 0.76 (5-year); accuracy = 70.3%; minimum sensitivity = 67.5%; logistic regression used for interpretability; decision rules generated for allocation support.	Prediction of graft survival to guide donor–recipient matching in organ allocation.
Li C et al. [64] (United States, 2024)	Retrospective study using SRTR dataset (2002–2021); 5-fold cross-validation.	*n* = 129,917 LT recipients.	FERI, logistic regression, DeepSurv, CPH; input: 49 pre-LT features (e.g., diagnosis, MELD, functional status, waitlist time).	Head-to-head comparison of FERI with DeepSurv, CPH, and logistic regression for graft failure prediction.	Model development only (retrospective).	Graft failure post-LT; FERI: AUC-ROC = 0.765; AUC-PR = 0.392; DPD = 0.125; EOD = 0.041; fairness-accuracy trade-off addressed via loss rebalancing.	Prediction of graft failure to improve equitable risk assessment in organ allocation.
Li C et al. [65] (United States, 2024)	Retrospective study using OPTN/UNOS transplant records (1987–2018); 5-fold cross-validation; 20% holdout test set.	*n* = 160,360 LT recipients.	Multi-task TabTransformer with task balancing and fairness-achieving algorithm vs. baseline single- and multi-task models; input: 52 recipient and 65 donor pre-LT variables.	Head-to-head comparison of task-balancing and fairness-optimized multi-task models vs. baseline methods.	Model development only (retrospective).	Post-LT complications (malignancy, DM, rejection, infection, and cardiovascular); fairness-optimized TabTransformer: AUC-ROC = 0.7315 (malignancy), 0.6600 (cardiovascular); AUC-PR range = 0.0753–0.3903; DPD and EOD reduced across gender, age, and race subgroups.	Prediction of malignancy, DM, rejection, infection, and cardiovascular complications post-LT.

**Table 4:** Contextual publications informing methodological approaches and evidence gaps in artificial intelligence applications for liver transplantation.

Study Identification	Study Type	AI/ML Focus	Transplant Phase	Targeted Outcome(s)	Methodological Highlights	Key Findings	Limitations/Gaps Identified
Chongo G et al. [66] (United Kingdom, 2024)	Systematic review	ML models including RF, GBM, DNN, ANN, SVM, and ensemble classifiers for mortality and complication prediction.	Pre- and post- transplant	Short- and long-term mortality, sepsis, AKI, GVHD, graft failure, and post-transplant HCC recurrence.	Comparison of RF, XGBoost, DNN, ANN, SVM, and LR model architectures across 23 studies; analysis of input features and AUC-ROC performance; benchmarking against MELD, D-MELD, BAR, SOFT, ABIC, and CLIF-based scores.	ML models consistently outperformed traditional prognostic scores across studies; RF and GBM demonstrated superior performance for 90-day mortality, sepsis, and AKI; DL models showed improved prediction for recurrence and long-term outcomes.	Predominance of retrospective designs, lack of standardization in model validation and input features, limited external validation, and underrepresentation of pediatric and low-resource settings.
Pruinelli L et al. [67] (United Kingdom, 2025)	Systematic review	Supervised (e.g., RF, ANN, and SVM), unsupervised (e.g., k-means and PCA), and DL models for predictive analytics, decision support, and workflow optimization.	Pre- and post- transplant	Graft survival, mortality, waitlist outcomes, rejection, infection, workflow efficiency, and decision support.	Comparison of supervised (RF, ANN, and SVM), unsupervised (k-means and PCA), and DL architectures across 68 studies; categorization of use cases (e.g., prediction, risk stratification, and clinical decision support); evaluation of model performance metrics (e.g., AUC-ROC and accuracy); thematic synthesis across clinical and operational domains.	AI applications demonstrated high predictive accuracy and potential for workflow integration; supervised learning dominated the field; growing use of multimodal data inputs and emphasis on clinical interpretability.	Heterogeneity in study designs and reporting standards; limited prospective validation; underuse of unsupervised methods; few studies addressed real-time clinical implementation.
Rahman MA et al. [68] (United Kingdom, 2023)	Systematic review	ML and DL models including SVM, RF, LR, CNN, LSTM, and ensemble methods for mortality and complication prediction in LT and hepatology.	Pre- and post- transplant	Graft and patient survival, liver disease progression, fibrosis staging, hospital readmission, infection, length of stay, and HCC recurrence.	Comparison of SVM, RF, LR, CNN, LSTM, and ensemble architectures; categorization of input variables (e.g., demographics, laboratory, imaging, and histopathology); evaluation of model performance using AUC-ROC, sensitivity, and specificity; integration of explainability techniques and bias mitigation approaches.	ML/DL models demonstrated superior performance over traditional statistical methods in predicting graft failure, fibrosis progression, and post-transplant complications; CNN and LSTM models showed enhanced accuracy for imaging and temporal data; studies incorporating model interpretability and fairness showed greater clinical applicability.	Limited external validation, heterogeneous input features and outcomes, low transparency of DL models, insufficient reporting of calibration, and lack of implementation studies.
Wingfield L et al. [69] (United Kingdom, 2020)	Systematic review	ML models including SVM, ANN, RF, decision trees, and LR for classification and prediction in solid organ transplantation.	Pre- and post- transplant	Graft survival, acute rejection, organ discard, and donor–recipient compatibility.	Analysis of ML model architectures (SVM, ANN, RF, LR, and decision trees); comparison of input features and classification tasks; discussion of model accuracy and application scope in LT.	ML models improved predictive performance for graft survival, rejection, and donor–recipient matching; ANN and SVM were most commonly used in LT studies; emphasized potential for AI integration into clinical decision-making.	Lack of transparency in model reporting, inconsistent performance metrics, low external validation, and underutilization of ML in liver transplantation compared to other solid organs.
Bhat M et al. [11] (Canada, 2023)	Narrative review	ANN, RF, GBM, SVM, and DL models across transplant domains.	Pre- and post- transplant	Graft and patient survival, waitlist mortality, acute rejection, HCC recurrence, fibrosis, metabolic complications (e.g., NODAT or cardiovascular disease), infection risk, and AKI.	Comparison of ANN, RF, GBM, and SVM architectures across over 60 studies; analysis of input variables (e.g., laboratory, clinical, imaging, and omics data); evaluation of model performance using metrics, such as AUC-ROC and cross-validation approaches.	AI-based models demonstrate higher discriminative performance than traditional statistical tools in predicting key outcomes; emphasis on integration of multimodal data (clinical, imaging, histologic, and omics) for improved post-LT management.	Lack of prospective validation, limited interpretability of models, underrepresentation of minority groups, incomplete data standardization, regulatory challenges, and absence of benchmarking frameworks.
Calleja Lozano R et al. [70] (Spain, 2022)	Narrative review	ANN and RF in donor–recipient matching and post-transplant risk stratification.	Pre- and post- transplant	Graft survival, donor–recipient compatibility, AKI, waitlist mortality, and post-transplant complications.	Comparison of ANN and RF architectures across three key studies; analysis of input variables (e.g., donor/recipient characteristics, MELD, BAR, and SOFT); external validation of MADRE model with KCH dataset and AUC-ROC performance benchmarking.	Higher predictive performance of ANN-based models (e.g., MADRE) compared to MELD, BAR, and SOFT scores in graft survival prediction; improved discrimination for donor–recipient compatibility; support for developing regionally adapted ANN frameworks to enhance transplant outcomes.	Limited generalizability, small and incomplete datasets, dependence on rules-based models, concerns related to algorithm transparency and ethical accountability.
Ferrarese A et al. [71] (Italy, 2021)	Narrative review	ANN, RF, Bayesian networks, SVM, classification trees, and DNN applied to survival modeling, organ allocation, and complication prediction.	Pre- and post- transplant	Waitlist mortality, post-LT survival, graft failure, HCC recurrence, AKI, acute rejection, NODAT, and early graft dysfunction.	Comparison of ANN, RF, DNN, SVM, Bayesian networks, and classification trees across multiple studies; analysis of model inputs and outputs; review of validation methods and integration into clinical workflows	ML models outperform traditional scores (e.g., MELD) in multiple outcome domains; ANN and classification trees show promise for donor–recipient matching and survival prediction.	Predominantly retrospective designs, limited external validation, insufficient standardization in model input/output, interpretability concerns, and lack of regulatory guidance.
Fuchs J et al. [72] (Germany, 2024)	Narrative review	ML and DL models including RF, Bayesian networks, LASSO, ridge regression, and CURATE.AI applied to pediatric LT outcomes.	Pre- and post- transplant	Waitlist mortality, acute liver failure prognosis, graft failure, rejection, ideal long-term outcomes, and tacrolimus dosing.	Comparison of RF, DL, Bayesian networks, ridge regression, LASSO, and CURATE.AI architectures across 8 pediatric LT studies; analysis of variables, including CYP3A5 genotype, GRWR, bilirubin, surgical parameters, and complications; evaluation of predictive accuracy across clinical use cases.	AI models show potential to enhance donor–recipient matching, outcome prediction, and personalized immunosuppression; RF and integrative models demonstrate promising accuracy in pediatric-specific cohorts.	Small sample sizes, retrospective designs, absence of prospective validation, low model interpretability, limited data standardization, ethical concerns, and lack of clinical implementation.
Gulla A et al. [73] (Lithuania, 2024)	Narrative review	ANN, RF, DNN, SVM, LR, and decision trees applied to liver graft survival prediction.	Post-transplant	Short-term and long-term graft survival.	Comparison of ANN, RF, DNN, SVM, and LR architectures across 17 studies; analysis of input variables (e.g., age, BMI, MELD, INR, and DM); AUC-ROC metrics across different model types.	RF and ANN models most frequently used; key predictive variables include recipient age, BMI, serum creatinine, INR, DM, and MELD score; AI models offer superior predictive performance over traditional scores.	Limited number of eligible studies; exclusion of HCC recurrence and donor–recipient matching models; lack of standardization in model input selection and validation metrics.
Ivanics T et al. [74] (Canada, 2020)	Narrative review	ML applications including ANN, RF, SVM, CART, KNN, and DL applied to transplant oncology.	Pre- and post- transplant	Post-LT HCC recurrence, overall and disease-free survival, microvascular invasion, tumour aggressiveness, and graft prioritization.	Comparison of ANN, RF, SVM, CART, KNN, and DL models across multiple studies in transplant oncology; analysis of input variables (e.g., AFP, tumour burden, genomics, radiomics, and pathology); performance metrics, including AUC-ROC, C-index, and concordance rates.	ML models demonstrated higher predictive accuracy than traditional clinical criteria (e.g., Milan, AFP, and MORAL); integration of imaging, genomic, and clinical data improved outcome prediction and organ allocation fairness.	Limited validation of most models, risk of overfitting, lack of standardization across data domains, reliance on retrospective datasets, and absence of causal inference capability.
Taner T et al. [75] (United States, 2022)	Narrative review	ML algorithms integrated with RNA-based and transcriptomic assays to improve rejection prediction and immunosuppression personalization.	Post-transplant	Acute cellular rejection, antibody-mediated rejection, operational tolerance, and immunosuppression modulation.	Comparison of ML-assisted transcriptomic and molecular assays across INTERLIVER and MAPLE studies; analysis of input variables (e.g., biopsy-derived RNA, blood-based biomarkers, and dual-miRNA panels); evaluation of model performance using AUC-ROC and correlation with rejection phenotypes.	ML-driven models enhance early rejection detection and support immunosuppression withdrawal strategies; AI integration improves risk stratification and immune monitoring in LT recipients.	Lack of clinical validation for composite biomarkers; limited access to high-throughput molecular assays; challenges in data harmonization, cost, and standardization.
Jiang L et al. [76] (China, 2025)	Methodological paper	Latent Dirichlet Allocation (LDAL)-based topic modeling and bibliometric analysis of artificial intelligence applications in acute rejection research.	Post-transplant	Acute cellular rejection, personalized immunosuppressive therapy, and molecular diagnostics.	Comparison of topic modeling and bibliometric mapping techniques across 1399 studies; analysis of input variables (e.g., miRNA, mRNA, cell-free DNA, donor-specific antibodies, and immunosuppressive strategies); implementation of VOSviewer, (v1.6.18), CiteSpace (v6.1.3) and R-bibliometrix (R v4.2.1) for cluster evolution and citation network mapping.	Transition in acute rejection research from histopathologic diagnostics to AI-integrated molecular profiling; rising emphasis on noninvasive biomarkers (e.g., donor-derived cfDNA, and miRNAs), immune tolerance, and personalized immunosuppressive strategies; identification of future hotspots including microbiome and regenerative therapies.	Reliance on publication metadata without clinical validation; underrepresentation of non-indexed studies; absence of standardized ontologies for topic classification; no patient-level data integration.
Khorsand SE et al. [77] (United Kingdom, 2023)	Commentary	DL (weighted LSTM networks) compared to conventional ML models (RF, SVM, logistic regression, LASSO, and ridge regression).	Post-transplant	F2 or greater graft fibrosis after LT.	Comparison of weighted LSTM with classical ML models and other DL architectures (RNN and TCN); analysis of 167,091 longitudinal data points across 1893 recipients; evaluation using Integrated Gradients, APRI, FIB-4, and transient elastography.	Weighted LSTM model outperformed traditional ML and DL models in predicting significant graft fibrosis; model captured temporal variability across follow-up intervals and showed consistent performance across disease etiologies and transplant eras.	Absence of validated reference standard for fibrosis in the transplanted liver; use of METAVIR score adapted from non-transplant biopsies; lack of prospective clinical validation and uncertainty in decision-making utility.

## Data Availability

The data supporting the findings of this study are available from the corresponding author upon request.

## Supplementary Materials

The following supporting information can be downloaded at: https://www.mdpi.com/article/10.3390/jcm15041491/s1/. Data S1: Complete search syntax for PubMed, Web of Science, and Cochrane.

## Abbreviations

The following abbreviations are used in this manuscript:

ABIC	Age–bilirubin–INR–creatinine
ACLF	Acute-on-chronic liver failure
AdaBoost	Adaptive boosting
AFP	Alpha-fetoprotein
AI	Artificial intelligence
AJCC	American Joint Committee on Cancer
AKI	Acute kidney injury
ALP	Alkaline phosphatase
ALT	Alanine aminotransferase
AMAE	Average mean absolute error
ANN	Artificial neural network
APRI	Aspartate aminotransferase to platelet ratio index
APTT	Activated partial thromboplastin time
AST	Aspartate aminotransferase
AUC-PR	Area under the precision–recall curve
AUC-ROC	Area under the receiver operating characteristic curve
AUSCAD	Australian chronic allograft dysfunction study
BAR	Balance of risk
BCLC	Barcelona clinic liver cancer staging system
BiGRU	Bidirectional gated recurrent unit
BMI	Body mass index
C5.0	Decision tree algorithm based on the C4.5 model, used for classification tasks
CART	Classification and regression tree
cDCD-NRP	Controlled donation after circulatory death under normothermic regional perfusion
cfDNA	Cell-free deoxyribonucleic acid
C-GBS	Component-wise gradient boosted survival
CI	Confidence interval
C-index	Concordance index
CIT	Cold ischemia time
CNN	Convolutional neural network
CoD-MTL	Cause-of-death multi-task learning
Cox-P	Cox model-based feature selection using p-values
CoxPH	Cox proportional hazards model
Coxnet	Cox proportional hazards model with elastic net regularization
CPH	Cox proportional hazards
CT	Computed tomography
DECIDE-AI	Developmental and exploratory clinical investigation of decision support systems driven by artificial intelligence
DeepSurv	Deep survival neural network
DL	Deep learning
DM	Diabetes mellitus
D-MELD	Donor age multiplied by recipient MELD score
DNN	Deep neural network
DPD	Demographic parity difference
DRI	Donor risk index
E-NET	Elastic net regression
EHCLT	European hepatocellular cancer liver transplant
EHRs	Electronic health records
EOD	Equalized odds difference
ERASL-post	European association for the study of the liver post-transplant model
ESLD	End-stage liver disease
Fair-ML	Fairness-aware machine learning
FERI	Fairness-enhanced risk index
FIB-4	Fibrosis-4 index
FS-SVM	Fast survival support vector machine
GBDTs	Gradient boosted decision trees
GBS	Gradient boosted survival
GMS	Geometric mean of sensitivities
GRWR	Graft-to-recipient weight ratio
GST	Glutathione s-transferase
GVHD	Graft-versus-host disease
HALT-HCC	Hazard associated with liver transplantation for hepatocellular carcinoma
HCC	Hepatocellular carcinoma
INR	International normalized ratio
i-RAPIT	Integrated radiology and pathology for immunotherapy-based transplantation
KCH	King’s College Hospital
KNNs	K-nearest neighbors
LASSO	Least absolute shrinkage and selection operator
LDA	Linear discriminant analysis
LDAL	Latent Dirichlet Allocation
LDLT	Living donor liver transplantation
LightGBM	Light gradient boosting machine
LR	Logistic regression
LSTM	Long short-term memory
LT	Liver transplantation
MACEs	Major adverse cardiovascular events
MADRE	Model for allocation of donor and recipient using artificial intelligence
MAPLE	Molecular assessment of predictive liver expression
MELD	Model for end-stage liver disease
MELD-Na	Model for end-stage liver disease with serum sodium
MELD 3.0	Updated model for end-stage liver disease, version 3.0
MeSHs	Medical subject headings
MIMIC-IV	Medical information mart for intensive care, version IV
miRNA	Micro ribonucleic acid
ML	Machine learning
MLP	Multilayer perceptron
MORAL	Model of recurrence after liver transplantation
mRNA	Messenger ribonucleic acid
MPENSGA2	Multi-objective evolutionary algorithm
NASH	Nonalcoholic steatohepatitis
NLP	Natural language processing
NODAT	New-onset diabetes after transplant
NPV	Negative predictive value
NRI	Net reclassification index
OPTN	Organ procurement and transplantation network
PCA	Principal component analysis
PND	Perioperative neurocognitive disorder
Post-LT	Post-liver transplantation
PPV	Positive predictive value
PRISMA-ScR	Preferred reporting items for systematic reviews and meta-analyses extension for scoping reviews
PROBAST-AI	Prediction model risk of bias assessment tool–artificial intelligence
PSC	Primary sclerosing cholangitis
PSSP	Patient-specific survival prediction
RBC	Red blood cell
RELAPSE	Recurrent liver cancer prediction score
ResNet-50	50-layer residual network
RETREAT	Risk estimation of tumor recurrence after transplant
RF	Random forest
RFE	Recursive feature elimination
Ridge	Regularized linear regression
RNA	Ribonucleic acid
RNN	Recurrent neural network
RSF	Random survival forest
SFM	Select from model
SHAPs	Shapley additive explanations
SOFA	Sequential organ failure assessment
SOFT	Survival outcomes following liver transplantation
SPLIT	Studies of pediatric liver transplantation
SpO_2_	Peripheral capillary oxygen saturation
SRTR	Scientific registry of transplant recipients
STAR	Standard transplant analysis and research
SVM	Support vector machine
TabNet	Tabular neural network
TCGA	The cancer genome atlas
TCNs	Temporal convolutional networks
TM-GTP	Tissue microdissection–genotype tissue profiling
TNM	Tumor–node–metastasis staging system
TOP	Transferable omics prediction
TRIPOD-AI	Transparent reporting of a multivariable prediction model for individual prognosis or diagnosis–artificial intelligence
U-net	U-shaped convolutional neural network
UCSF	University of California San Francisco
UHN	University health network
UMHTC	US Multicenter HCC Transplant Consortium
UNOS	United Network for Organ Sharing
WBC	White blood cell
WIT	Warm ischemia time
XGBoost	Extreme gradient boosting
