# Supplementary material for: Artificial Intelligence in Post-Liver Transplantation: A Scoping Review of Comparative Model Performance
Source: J Clin Med. 2026 Feb 13;15(4):1491. doi: 10.3390/jcm15041491 (PMC12941496; doi:10.3390/jcm15041491)
Supplement: Supplementary file 1 [file jcm-15-01491-s001.zip › jcm-4129897-supplementary.pdf]

PubMed

("artificial intelligence"[MeSH Terms] OR "artificial intelligence"[All Fields] OR "machine learning"[MeSH Terms] OR "machine learning"[All Fields] OR "deep learning"[All Fields] OR "neural network*"[All Fields] OR "natural language processing"[All Fields] OR "predictive model*"[All Fields] OR "algorithm*"[All Fields]) AND ("liver transplantation"[MeSH Terms] OR "liver transplant*"[All Fields] OR "hepatic transplant*"[All Fields] OR "liver graft*"[All Fields]) AND ("workflow*"[All Fields] OR "decision support"[MeSH Terms] OR "decision support"[All Fields] OR "allocation"[All Fields] OR "matching"[All Fields] OR "prediction"[All Fields] OR "risk stratification"[All Fields] OR "monitoring"[All Fields] OR "complication*"[All Fields] OR "survival"[All Fields] OR "outcome*"[All Fields] OR "resource utilization"[All Fields] OR "efficiency"[All Fields])

Web of Science

TS=("artificial intelligence" OR "machine learning" OR "deep learning" OR "neural network*" OR "natural language processing" OR "predictive model*" OR algorithm*) AND TS=("liver transplant*" OR "hepatic transplant*" OR "liver graft*") AND TS=(workflow* OR "decision support" OR allocation OR matching OR prediction OR "risk stratification" OR monitoring OR complication* OR survival OR outcome* OR "resource utilization" OR efficiency)

Cochrane

#1 MeSH descriptor: [Artificial Intelligence] explode all trees
#2 ("artificial intelligence"):ti,ab,kw
#3 ("machine learning"):ti,ab,kw
#4 ("deep learning"):ti,ab,kw
#5 ("neural network*"):ti,ab,kw
#6 ("natural language processing"):ti,ab,kw
#7 ("predictive model*"):ti,ab,kw

#8 (algorithm*):ti,ab,kw

#9 #1 OR #2 OR #3 OR #4 OR #5 OR #6 OR #7 OR #8

#10 MeSH descriptor: [Liver Transplantation] explode all trees

#11 ("liver transplant*"):ti,ab,kw

#12 ("hepatic transplant*"):ti,ab,kw

#13 ("liver graft*"):ti,ab,kw

#14 #10 OR #11 OR #12 OR #13

#15 ("workflow*"):ti,ab,kw

#16 MeSH descriptor: [Decision Support Techniques] explode all trees

#17 ("decision support"):ti,ab,kw

#18 ("allocation"):ti,ab,kw

#19 ("matching"):ti,ab,kw

#20 ("prediction"):ti,ab,kw

#21 ("risk stratification"):ti,ab,kw

#22 ("monitoring"):ti,ab,kw

#23 ("complication*"):ti,ab,kw

#24 ("survival"):ti,ab,kw

#25 ("outcome*"):ti,ab,kw

#26 ("resource utilization"):ti,ab,kw

#27 ("efficiency"):ti,ab,kw

#28 #15 OR #16 OR #17 OR #18 OR #19 OR #20 OR #21 OR #22 OR #23 OR #24 OR #25 OR #26 OR #27 #29 #9 AND #14 AND #28
